# Update on the Improving Access to Psychological Therapies programme in England^†^: Commentary on … Children and Young People's Improving Access to Psychological Therapies

**DOI:** 10.1192/pb.bp.115.052282

**Published:** 2015-10

**Authors:** Peter Fonagy, David M. Clark

**Affiliations:** 1 University College London, London; 2 University of Oxford, Oxford

## Abstract

Professor Sami Timimi recently expressed concerns about the Improving Access to Psychological Therapies (IAPT) programme. We argue that the concerns are largely unfounded and provide readers with an update on the programme.

We are pleased to have the opportunity to respond to Professor Timimi's editorial in the April 2015 issue of *BJPsych Bulletin* and his comments on the Improving Access to Psychological Therapies (IAPT) programme in general and the Children and Young People's Improving Access to Psychological Therapies (CYP-IAPT) programme in particular. We hope to point out that many of his concerns about both programmes are unfounded, either because evidence we have collected should allay his concerns or because the issues he identifies represent misunderstandings of the literature and the initiatives.

## Adult IAPT

The IAPT programme for adults aims to make National Institute for Health and Care Excellence (NICE)-recommended psychological treatments for depression and anxiety disorders more widely available by training 6000 new psychological therapists and deploying them in specialised stepped-care services, along with experienced staff. The initiative has several distinctive features, which an editorial in the journal *Nature* described as ‘world-beating’.^1^ For the first time, therapist training follows nationally agreed curricula that focus on the competencies required to deliver those treatments that have been shown by randomised controlled trials (RCTs) to be effective for particular conditions. An innovative session-by-session outcome monitoring system captures clinical outcomes on almost everyone (97%) who has finished a course of treatment (liberally defined as at least two sessions, although many people have more),^2^ and clinical commissioning group (CCG)-level outcome data are published on a public website.^3^ This contrasts sharply with the picture before IAPT, when psychological therapy services obtained outcome data on less than 40% of patients and are likely to have overestimated their value, as people whose data are missing tend to have done less well.^4^

What does the more complete IAPT data tell us? Nationally, of patients who have finished a course of treatment in IAPT, 45% recover (based on a strict double criterion – dropping below the clinical threshold for both anxiety and depression) and a further 16% show reliable improvement that falls short of full recovery.^2,5^ However, there is considerable regional variability. About a third (70 of 211) of CCGs now report recovery over 50% and some are consistently over 60% (www.hscic.gov.uk). This shows what the IAPT model can achieve in services with a sufficiently large and appropriately trained workforce that benefits from excellent clinical leadership. The challenge for the next phase is to raise other CCGs to the same level.

Professor Timimi's editorial^6^ asserts that there is little evidence for the premises on which adult IAPT is based, and that IAPT services are less effective and more expensive than pre-existing counselling services. Both assertions can be refuted.

### IAPT outcomes

IAPT delivers therapies that have been shown to be effective for particular conditions in RCTs, on the assumption that this is the best way of ensuring good clinical outcomes. Timimi argues this is mistaken because ‘within treatment, the factor that has the biggest impact on outcomes is the therapeutic alliance (as rated by the patient) with matching treatment model to diagnosis having a small to insignificant impact’.^6^ Contrary to this statement, there is evidence in all anxiety disorders that some psychological treatments are more effective than others.^5^ In depression, the picture is less clear. Several psychological therapies have been shown to be better than placebo or no treatment, but there is little evidence of differential effectiveness between these therapies.^5^ However, there is no such thing as a therapeutic alliance therapy. Even if therapeutic alliance were the most important factor, one would still need to train therapists in procedures that allow the therapeutic alliance to emerge. Clearly, it makes sense to choose procedures from treatments that are known to work. It is also important to note that the use of weak research designs means that many studies of therapeutic alliance are likely to overestimate the importance of this factor. Alliance is often measured late in therapy when some patients have already improved. The correlation between alliance and outcome may therefore be a consequence, rather than a cause, of clinical improvement. Feeley's studies^7^ showing that late alliance is related to outcome but early alliance is not are consistent with this point of view. Alliance studies rarely measure the competence with which a treatment is delivered, so we cannot rule out the possibility that positive alliance ratings reflect competent and sensitive delivery of a treatment, not just the establishment of a good therapist–patient relationship. The remarkable success of internet-delivered therapies strongly challenges the claim that a strong therapeutic alliance is essential.

The report^8^ that Professor Timimi cites as evidence that IAPT services are less effective and more costly than pre-IAPT counselling services is flawed. It was produced by a charity that funded some pre-IAPT counselling services and does not appear to have gone through a normal peer-review process. It fails to describe its methods and measures in the level of detail required for a journal article. However, from the details that are available, it is clear that the report is comparing chalk with cheese. IAPT recovery rates were based on all patients who had at least two sessions. Pre-IAPT services had high post-treatment missing data rates^4^ and recovery rates were based on those who contributed post-treatment data, which will inevitably inflate estimates.^9^ The IAPT recovery criterion was a strict double-measure criterion, whereas pre-IAPT recovery was based on a more lenient, single-measure criterion. Costs for IAPT and non-IAPT services were also estimated differently, with the former including set-up, staff training and premises costs, while it seems unlikely these figured in the comparator costs.^8^

The characterisation of IAPT as a ‘fetishisation’ of cognitive-behavioural therapy (CBT) is also misplaced. As NICE only recommends CBT for anxiety disorders and depression, the initial focus of IAPT was on this modality. However, the programme now supports the training and employment of therapists who can deliver the four other therapies that NICE recommends for depression (counselling, couples therapy, interpersonal psychotherapy and brief psychodynamic therapy). Counselling is already strongly represented, by over a quarter of all IAPT high-intensity therapists. There is a need to build further capacity in the other three therapies and this is already underway. In the past 2 years IAPT has trained more therapists in non-CBT modalities than in CBT.

## Children and Young People's IAPT

Children and Young People's IAPT (CYP-IAPT) is training many more systemic family practitioners, interpersonal psychotherapists and parenting therapists than CBT therapists, and our core curriculum has made use of client feedback to inform practice across modalities. Professor Timimi's emphasis on therapeutic alliance is consistent with CYP-IAPT's fundamental concern with collaborative practice and shared decision-making.

Professor Timimi's first concern about CYP-IAPT is the risk of a top-down *v*. bottom-up approach to service transformation. He suggests that rather than using research evidence to guide selection and implementation of interventions, it would be cheaper and better for child and adolescent mental health services (CAMHS) simply to roll out usual care based on his own Partners for Change Outcome Management Systems (PCOMS) model for service transformation. He cites a selection of meta-analyses to suggest that interventions targeted at specific disorders have no effect, even when we know RCTs indicate large effect sizes. Comparisons of psychological therapies with usual care indeed tend to have small effects but this depends entirely on the services offered in usual care. For example, multisystemic therapy has very large effects because the usual care comparator is often part of youth justice provision.^10^ Comparisons with community-based active treatments yield far smaller effects. Many advocates of evidence-based therapies (EBTs) have pointed this out,^11^ and there are indications that modular-based approaches integrating a range of EBT elements may well be the way forward.^12^ On the whole, comparisons with usual care show the difficulty of designing new interventions that systematically outperform the old.^13^ But does this warrant complacency about usual care in CAMHS? Existing evidence for the effectiveness of ordinary CAMHS in the UK and elsewhere should worry both clinicians and policy makers. The observed effect sizes are small and sometimes even statistically insignificant. The majority of children receiving community-based usual care do not show clinical improvement.^14,15^

Unfortunately, bringing about improvements appears quite challenging. In his editorial Professor Timimi cites the historical Stark County and Fort Bragg studies, which are relevant to this debate for two reasons. First, they highlighted the importance of the method of implementation in service improvements. When major service improvement initiatives are launched, evidence now has to be presented that implementation science principles are followed. For example, organising observation of clinical work as part of supervision is essential for rigorous training of therapists,^16^ as is the structuring of services to accommodate EBTs.^12^ Second, the two studies led implementers and others to stress the likely importance of continuous, clinically meaningful feedback and progress monitoring. Implementation science considerations and meaningful use of session-by-session outcome measurement have informed CYP-IAPT's work from the beginning.^17^ Rather than seeking to impose a single solution on all services, we have tried to disseminate a set of clinical principles (outcome focus, increased patient and parent participation, use of EBTs) and to implement these through local collaborations engaging a range of services, which jointly tried to find the best way forward.

## CYP-IAPT: a model for child mental health services

There is no template for a CYP-IAPT service; there are services that use CYP-IAPT principles. PCOMS, or any other service model, could only be universally implemented as part of a top-down initiative. As Professor Timimi highlights, this was precisely what went wrong at Fort Bragg and Stark County, and we did not wish to repeat the error. As a national programme, CYP-IAPT required a modest governance structure, including focused work streams to develop curricula, outcomes measurement and service organisation, but it was delivered within CAMHS partnerships made up of commissioners and both statutory and non-statutory providers who wanted to deliver local change. Through a well-recognised (evidence-based) phasing of the change process (exploration, installation, initial implementation, innovation and sustainability), we engaged services covering 68% of the population in the 1–19 years age bracket within 4 years. Although we may not have succeeded everywhere, our explicit strategy was to mobilise the local leadership and workforce to engage children/young people and their parents in the process of service transformation, including – but reaching out beyond – the National Health Service (NHS), to achieve sustainable results.

A recent benchmarking survey found that 70% of CAMHS questioned said they were working to CYP-IAPT principles.^18^ According to the annual update of data submitted for CYP-IAPT, data completeness of matched cases at time 1 and time 2 cases was 63.2%.^19^ The Rapid Internal Audit^20^ of 12 representative partnerships, over 350 clinicians and several focus groups of children and parents/carers found that:

1. The percentage of cases closed by mutual agreement out of all closed cases has increased by 75% since the initiation of CYP-IAPT.
2. The number of weeks between referral and first appointment has decreased from 16.6 in year 1 of CYP-IAPT to 6.6 in year 5.
3. The proportion of self-referrals, although still relatively small, increased by 51%.
4. 54% of clinicians agreed that the service was working towards the principle of increased self-referral; 61% agreed that access had improved for their local population over the past year.
5. Well over half of clinicians questioned reported often or always using outcome data to review treatment progress or to inform therapy; a similar percentage reported often or always discussing outcomes data with children/young people and families.
6. More than three-quarters of clinicians reported usually or always engaging in shared decision-making activities with parents/carers and children.
7. Children in focus groups spoke spontaneously about how their involvement in service delivery gave them a personal sense of worth and empowerment. Children and young people also agreed that monitoring outcomes helped to keep things focused.

Professor Timimi identified delivering EBTs as the sole objective of CYP-IAPT. In fact, we set ourselves six evidence-based objectives to improve services for children and young people (www.cypiapt.org/children-and-young-peoples-project.php). In addition to (1) delivering EBTs, we aimed to (2) improve access through self-referral, (3) work in partnership with the young person and their parent or carer throughout treatment, (4) deliver outcomes-focused treatments, (5) provide supervision to support delivery of evidence-based, user- and outcomes-informed practice, and (6) support whole-service transformation through leadership training. As we understand PCOMS' priorities, these have much in common with the CYP-IAPT curricula: the emphasis on consultation, involving optimal collaboration with other agencies; outcomes focus, using session-by-session patient-rated outcome data and changing treatment if outcomes are not improving; developing effective treatment alliances aided by the outcomes focus; developing team cultures that are recovery focused; and understanding how to use outcome data for clinical reflection, supervision and whole-team development.

CYP-IAPT insists on the use of treatment protocols based on manuals validated by one or more RCTs. Delivery of these protocols requires a clear set of competencies, which therapists must show they possess. Professor Timimi is committed to the common factors model of therapeutic change, and believes that generic therapeutic competencies are sufficient to deliver effective help regardless of the nature of the child's disorder. Although many therapies share important elements, such as a strong therapeutic relationship, researchers have found that not all therapies work equally well for all childhood disorders.^21^ Some therapies have actually been shown to be harmful.^22^ In the case of conduct problems, anxiety-related diagnoses (e.g. generalised anxiety disorder and obsessive-compulsive disorder), attention-deficit hyperactivity disorder and a number of other disorders, there is clear evidence supporting skilled manualised interventions, which could not be accounted for by common factors such as the therapeutic alliance.^21^ We also know from implementation science that attending training workshops is not sufficient to acquire competence in an intervention.^16^ The CYP-IAPT training includes intensive workshops and ongoing supervision/consultation, including practice sample review (e.g. audiotape review).

During the earliest exploration phase of the implementation process, the Department of Health and then NHS England sought to learn from local providers to build on existing best practice rather than implementing from above. We established collaboratives based around higher education institutions to lead the implementation locally, as individuals, organisations and system units gained competence and confidence in the new ways of delivering therapy. In the current ‘innovation’ phase of CYP-IAPT, a national group, the Collaborative of Collaboratives, is presenting opportunities for CAMHS partnerships to refine and expand both the treatments and the implementation of the programme, and the group tasked with service transformation has drawn up a template for improved services (*Delivering With and Delivering Well*),^23^ co-authored by the Child Outcomes Research Consortium (CORC), the Quality Network for Community CAMHS (QNCC), the Choice and Partnership Approach (CAPA) and Youth Access, young people and other voluntary organisations, against which the quality of services can be judged.

Our current focus is the sustainability phase, which requires a national system of quality assurance of training, performance and service characteristics so that CAMHS partnerships can be held to account for maintaining the system they have established. An Accreditation Council – working in partnership with the Royal College of Psychiatrists, the British Psychological Society, NHS England, Health Education England, the QNCC, and the professional groups representing family therapists, interpersonal psychotherapists and CBT therapists – has developed an individual accreditation system for CYP-IAPT. These measures help commissioners and providers ensure that children, young people and parents receive the appropriate, evidence-based, outcomes-focused care they deserve.

That these improvements have been possible against the background of the most significant challenges across child mental health since the establishment of child guidance clinics 60 years ago is a testament to the incredible commitment to innovation of the CAMHS partnerships, their clinicians, leaders, the children, young people and parents, as well as the higher education institutions supporting their development. The high profile of children and young people's mental health has been boosted by the demonstration of effectiveness. We look forward to a brighter future for CAMHS, characterised by improved accessibility, more participation, an increased outcomes focus, greater transparency, and continued respect of NICE guidance and evidence-based practice.
